# Telesupervision of allied health students during work-integrated learning: a scoping review of the international evidence-base and its representation in localised professional guidelines

**DOI:** 10.1186/s12909-026-09440-z

**Published:** 2026-08-06

**Authors:** Catherine Easton, Lisa Brown, Melissa Nott, Narelle Patton, Elsie de Klerk, Therese Jones-Mutton, Skye Sewell

**Affiliations:** 1 Institute of Health and Wellbeing, Federation University, Berwick, VIC Australia; 2 Faculty of Science and Health, Charles Sturt University, Albury, NSW Australia; 3 Three Rivers Department of Rural Health, Charles Sturt University, Albury, NSW Australia; 4 Three Rivers Department of Rural Health, Charles Sturt University, Dubbo, NSW Australia; 5 Three Rivers Department of Rural Health, Charles Sturt University, Wagga Wagga, NSW Australia; 6 Queensland Department of Education, Brisbane, QLD Australia

**Keywords:** Allied health education, Telesupervision, Clinical supervision, Clinical placement, Scoping review

## Abstract

**Background:**

Work-integrated learning (WIL) plays a critical role in the experiential construction and assessment of allied health students’ professional capabilities, forming a core component of allied health education programs. Innovation in work-integrated learning has many social, economic, and technological drivers. In allied health education, telesupervision, a technology-mediated clinical supervision model, is one such innovation that aims to provide accessible WIL opportunities; however, there is a pressing need to assure the quality of telesupervision WIL experiences.

**Methods:**

This review integrated a scoping review of internationally published evidence (based on PRISMA-ScR) alongside a localised review of professional documents obtained from Australian allied health professions websites. This approach sought to synthesise the international evidence-base for telesupervision and understand how Australian allied health professions were applying this evidence to shape the education of future professionals. The review addressed three research questions: (1) What evidence exists to support and guide educational providers in assuring quality telesupervision for allied health students?; (2) What evidence exists regarding supervisors’ and allied health students’ experiences of telesupervision?; and (3) What professional requirements and/or information exists to support and guide the provision of telesupervision to allied health students by clinical supervisors in Australia?

**Results:**

Thirty three articles were included in the review. Study designs were mainly descriptive or qualitative and thus provided limited support for assuring quality telesupervision practice. Studies identified high satisfaction with telesupervision among clinical supervisors and students, highlighting its role in developing clinical skills, confidence, and telehealth-specific competencies. The professional document review revealed a lack of profession-based guidance for implementation of telesupervision, reflecting the insufficient evidence-base to assure quality telesupervision.

**Conclusions:**

Despite a limited evidence-base, experience studies suggest that telesupervision is a valuable work-integrated learning model for students and clinical supervisors. By overcoming geographical barriers and preparing students for contemporary healthcare practice, it offers professionally relevant opportunity for increasing accessibility of health professions education. Further research is needed to establish effectiveness of student learning through telesupervised work integrated learning and inform professional guidelines within localised contexts.

**Supplementary Information:**

The online version contains supplementary material available at https://doi.org/10.1186/s12909-026-09440-z.

## Background

Work-integrated learning (WIL), also known as co-operative education, service learning, internships, fieldwork, clinical placement, clinical education and practice education (CEWIL) [1], has long been a central pillar of university allied health education. In this study, WIL is defined as student participation in workplace learning or clinical placement in a healthcare or related setting. Through engagement in WIL, students are provided supervised opportunities to develop competencies required for practice by accrediting bodies [1–3]. Allied health WIL provision is highly regulated by professional accrediting bodies with stipulations relating to who supervises students, time required to develop competency, models of WIL employed, and the use of simulation [2]. Increasingly, universities are required to be innovative when designing and delivering WIL opportunities in response to changing tertiary, healthcare and societal needs, including changes to governance, regulation and funding models, changing student cohorts, the economic environment, improved interactive technologies.

Highlighted by responses to COVID-19, broader acceptance of online clinical education has enabled students to engage in allied health clinical education despite geographic barriers [4]. Telesupervision, defined here as a supervisory model through which the clinical supervisor provides profession specific or interprofessional supervision via telephone and/or online to an allied health student, offers flexible clinical placement opportunities where students, clinical supervisors, and clients are not geographically co-located [5]. Examples of clinical placements incorporating telesupervision are growing rapidly following increased need during the COVID-19 pandemic [6]. Other drivers for telesupervision are evident in areas of allied health workplace shortage, where the presence of qualified and registered allied health professionals to provide direct student supervision is limited. For example, telesupervision models have been adopted to overcome the geographical challenges related to allied health student placements in rural areas of Australia [7] and in developing countries with limited access to allied health professionals [8]. Thus, ongoing use of telesupervision post COVID-19 has potential for increasing the accessibility of clinical-placement experiences internationally.

With these drivers increasing the demand for allied health telesupervision practice, there is also pressing need to assure the quality of telesupervision in work integrated learning in response to the increased regulation of the higher education sector. This international trend is realised through documents such as national frameworks and guidelines. For example, in the Australian context, the Work-Integrated Learning Australia LTD (WIL Australia) endorsed framework to support assurance of institution-wide quality in work integrated learning requires design of work integrated learning experiences to be underpinned by research and scholarship (Standard 2.1) [9]. Additionally, health professional boards and associations who undertake curriculum review and accreditation (e.g., Speech Pathology Australia) also require work integrated learning practices to be supported by high quality evidence.

Thus, this study aimed to identify and synthesise the existing evidence-base for telesupervision during allied health clinical placements, regarding identification of ‘quality’ telesupervision of allied health students, as well as student and clinical supervisor experience. To situate the review of international peer-reviewed published literature within the context of practice, the researchers drew on their knowledge and experience of the Australian allied health professions and higher education sector, allowing for localised application and critique of findings.

Thus, the current study aimed to address the following three research questions:

1. What evidence exists to support and guide educational providers in assuring quality telesupervision for allied health students?
2. What evidence exists regarding supervisors’ and allied health students’ experiences of telesupervision?
3. What professional requirements and/or information exists to support and guide the provision of telesupervision to allied health students by clinical supervisors in Australia?

## Methods

The practice-based focus of this study led to the adoption of a pragmatist methodological framework [10]. Multiple methods were drawn upon to understand the current evidence-base represented in the literature, and professional publications that would typically be excluded from a standard literature review protocol. Thus, a scoping review of relevant peer-reviewed published literature was situated alongside a review of publicly available documents published by health professional organisations in Australia to better understand how Australian allied health professions are currently applying this information for the purposes of national accreditation. The method of each approach is provided below.

### Scoping review of published evidence

The Arksey and O’Malley framework for scoping reviews was adopted [11]. The Preferred Reporting Items for Systematic reviews and Meta-Analyses extension for Scoping Reviews (PRISMA-ScR) guidelines [12] were applied to systematically identify, screen, and select studies relevant to research questions 1 and 2.

#### Identification of relevant articles

Key terms were identified and discussed during research team meetings and reviewed in conjunction with the university librarian. Due to the variability of terms across disciplines and workplace contexts, the list of synonyms was trialed and refined multiple times before the final list was established. Key terms were defined as follows: *Allied health students* are students enrolled in a Bachelor or Master course; *Work Integrated Learning* is participating in workplace learning or clinical placement in a healthcare or related setting; and *Telesupervision* is a supervisory model through which the clinical supervisor provides profession specific or interprofessional supervision via telephone and/or online to an allied health student who is located remotely from the clinical supervisor, in configurations that include the client also being online, or being located with the student [5]. Based on these definitions, the PCC framework (population, concept, and context) [13] was used to generate the keywords in Table 1.

**Table 1:** PCC keywords and selected synonyms

Population: Allied health student(s)	Context: Work-integrated learning	Concept: Telesupervision
Allied health student/s Health student/s University Student/s Tertiary student/s	Workplace learning Clinical placement Work-integrated learning Clinical education Supervision Student supervision	Telesupervision Online supervision Online education Telehealth supervision Virtual supervision e-supervision

The following databases were searched in May 2022 and November 2023: CINAHL, EBSCOhost (Health, Education), MEDLINE, ProQuest (Health & Medicine, Nursing & Allied Health, Education), and ERIC. Authors SS, MN and CE refined the terms for each database through consultation with the university librarian. Search terms were applied individually and then combined using “OR” to link parallel concepts, followed by “AND” to link the three key search concepts. Truncation or wildcard characters were used (for example “supervis$” to include “supervisor” and “supervision”). Searches were restricted to articles published in English between 2003 and 2023, starting with the evolution of synchronous internet-based technologies and concluding with publications during and immediately following COVID-19. An example search strategy is included in Appendix 1.

Outputs from database searches were exported to one integrated EndNote [14] library then uploaded into Covidence [15], and duplicates removed. All screening and review steps were supported within Covidence [15]. All authors participated in title and abstract screening. Two authors independently reviewed each article, with a third reviewer used to resolve disagreements. Full text review of all remaining articles was conducted following the same protocol.

The following inclusion criteria were applied:

- Articles that referred to allied health students and a clinical supervisor engaging in online supervision for the purpose of providing clinical education to the student.
- Education context was clinical placement (including university-based health clinics and simulation).
- Peer-reviewed articles.
- All article types (including reviews).
- All countries of origin.

The following exclusion criteria were applied:

- Supervision of health professional graduates.
- Mentoring of health professional graduates or students.
- University based education context (unless it was a university-based health clinic).
- Articles written in languages other than English.
- Conference proceedings with only abstracts available.
- Protocols for reviews or randomised-control trials.

**Fig. 1:**
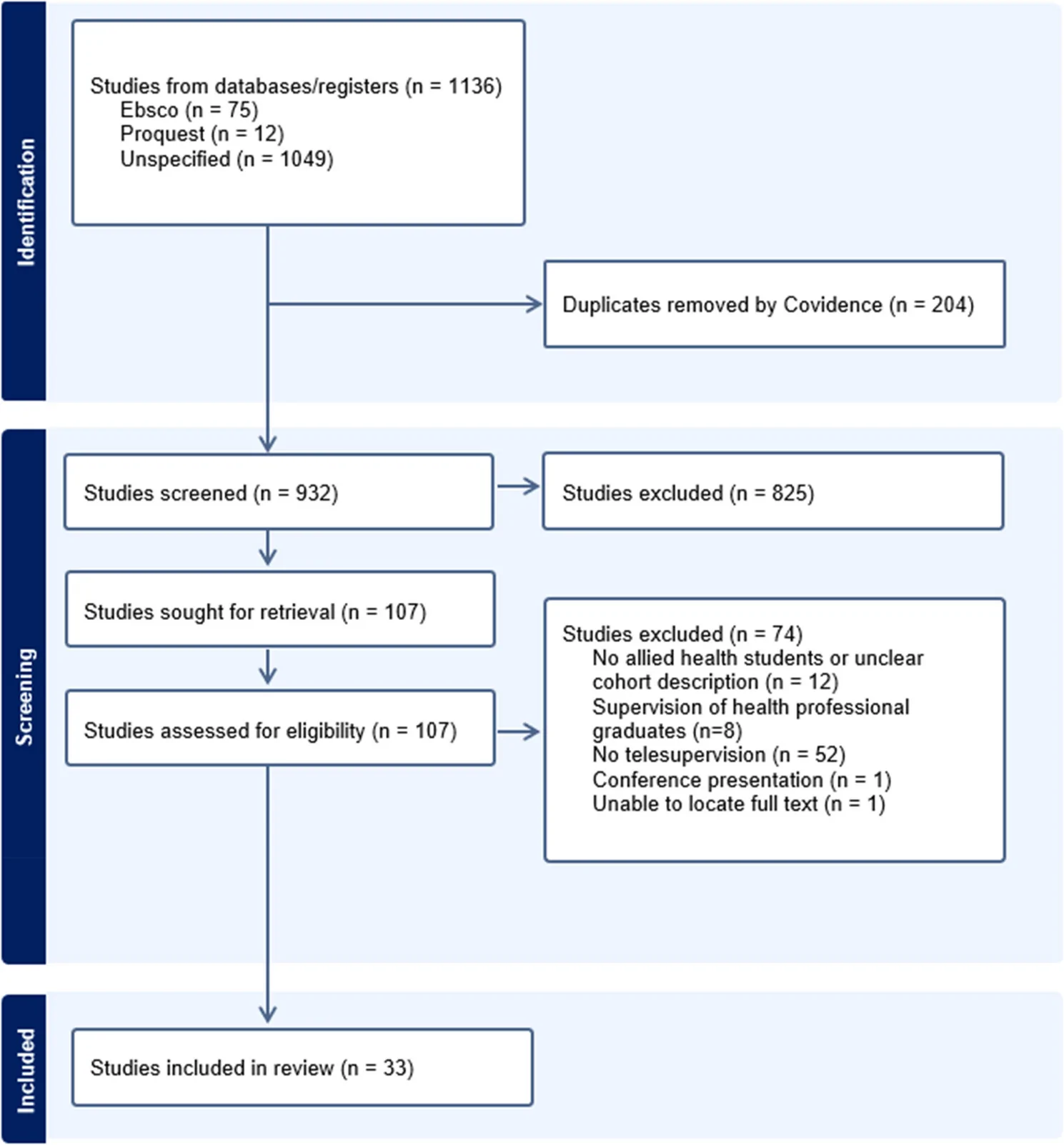
Preferred reporting items for systematic reviews and meta-analyses flow diagram (PRISMA)

This process is illustrated in Fig. 1.

#### Data extraction

All authors contributed to developing a data extraction template in Covidence [15]. Extracted study characteristics included: publication date; country; study design and aim/s; data collection methods; timing relative to COVID-19; and funding. Extracted characteristics of telesupervision were data driven, based on the researchers’ knowledge of the studies gained during screening. Domains included: location of student/s, clinical supervisor, and client/s; health discipline of clinical supervisors and students; reporting of interprofessional education or supervision; client characteristics and nature of health services provided. During the extraction process, reviewers also recorded quantitative and/or qualitative outcome data and a summary of study recommendations for future research or practice involving telesupervision. Two authors independently extracted data from each study using the extraction template. Disagreements were resolved by consensus.

#### Data analysis

Extracted data were tabulated. Study design, context, participants and other methodological data were extracted. Author MN collated quantitative outcome data and identified areas of overlapping outcome reporting. Three primary outcome areas were identified across studies. These included: telesupervision satisfaction; clinical practice and information technology (IT) skills development; and impact on sense of isolation. Data across all three primary outcome areas were analysed to determine the perspective from which outcome data was sought, student or supervisor.

Qualitative findings were analysed through inductive content analysis [16] of extracted data based on meanings in extracted data. Author LB uploaded the extracted qualitative findings into NVivo and completed initial data coding and code categorisation into categories and sub-categories. The research team reviewed initial coding and categorization and discussed the initial analysis to further enhance reliability and confirm category labels. Following this process, Author LB additionally coded data as “supervisor perspective” or “student perspective”. An NVivo Matrix Coding Query was applied to compare the “supervisor” and “student” perspectives across the categories and subcategories identified. An example table of categories, subcategories and examples from extracted data is provided in Appendix 2.

Recommendations for future research or practice involving telesupervision were similarly analysed [16]. Authors CE and ED identified emergent codes based on meanings in the extracted data and continued to refine these as analysis continued. A subsequent process of combining and dividing coded data to ensure shared understanding and meanings resulted in draft categories. Findings were discussed with the research team to enhance reliability and confirm category labels.

### Review of professional documents

The review of professional documents applied a systematic process to identify, screen, and select website information relevant to research question 3.

#### Key definitions and search terms

The primary keyword for the website search was *telesupervision* (as defined in Review of published evidence – Key definitions above). Secondary search terms: *clinical education*,* student supervision*,* student placement*,* telehealth*,* telepractice*,* and student learning*, were included.

#### Website search

Website searches of Australian allied health professions and supporting interprofessional organisations were undertaken. Inclusion criteria were allied health professions listed by the Australian Health Practitioner Regulation Agency (AHPRA, [17]) or the National Alliance of Self-Regulating Health Professions (NASRHP, [18]). Medicine, nursing, and midwifery were excluded. Interprofessional organisations known to the authors that support the work of allied health professions and/or allied health education in Australia were included. Twenty-three allied health professions and six supporting organisations were identified, and website addresses obtained. For AHPRA registered professions, the relevant board website was included. See Appendix 3 for a list of identified professional associations, boards, and supporting interprofessional organisations with website addresses.

#### Screening

**Fig. 2:**
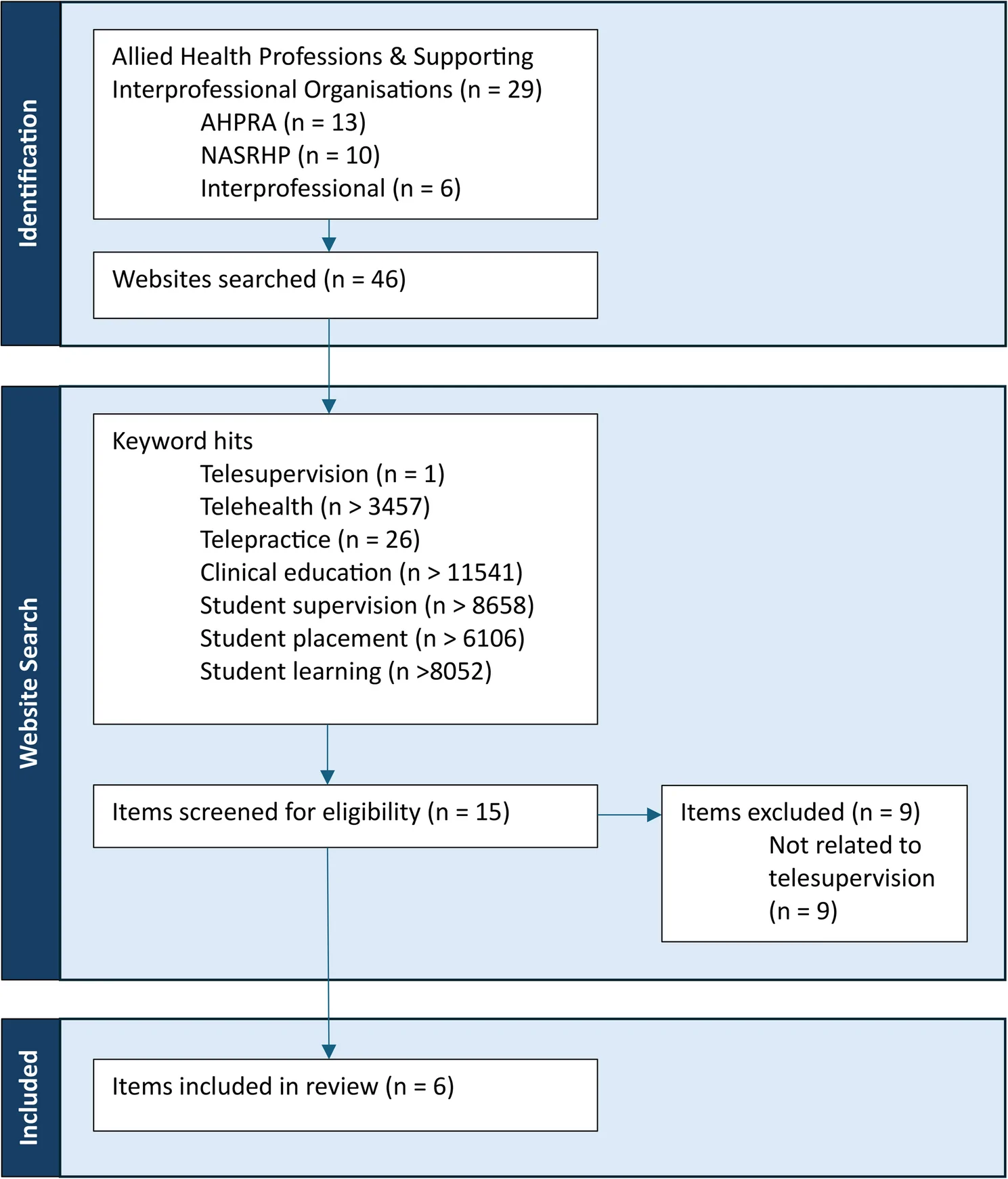
Professional document review data collection flowchart

Author LB searched the publicly accessible areas of each website for formal website content (i.e., in html format) and downloadable profession-based documents (e.g., position statements, policy documents, or similar) related to telesupervision. Each website’s search engine tool was used to identify results. Potentially relevant content and documents were visually reviewed and excluded based on relevance. If a website did not include a search engine tool, it was visually reviewed for any tabs or links related to each of the identified keywords. Results related to informal publications such as newsletters; brief opinion pieces; and advertisements for conferences, professional development activities, or scholarship opportunities were excluded. This process is detailed in Fig. 2.

#### Data extraction and synthesis

All documents identified as relevant were downloaded when possible, or detailed notes made about the telesupervision content. Author LB reviewed all website telesupervision content and profession-based documents to extract and summarise relevant data. Due to the small number of sources, a detailed analysis was not suitable.

## Findings

This section reports on the results of each of the research methods before the findings are synthesised to create an integrated response to the research questions in the discussion.

### Findings from published evidence

The review of the published literature enabled exploration of research questions 1 and 2. A total of 1136 articles were imported for screening. After removal of 204 duplicates, 932 articles were screened at abstract and title stage. 107 articles were included for full-text review. 74 articles were excluded resulting in 33 included articles (refer to Table 2).

**Table 2:** Characteristics of included articles

Study ID	Country	Study design	Perspective	Allied health discipline/s
Pre-COVID articles				
Chipchase et al. (2014) [19]	Australia	Mixed-methods	Supervisor	OT, Physio, SLP
Dudding & Justice (2004) [20]	US	Descriptive	-	SLP
Furness & Kaltner (2015) [21]	Australia	Mixed-methods	Student	OT
Miller et al. (2003) [22]	US	Qualitative	Supervisor	OT, Physio, Psychology, SLP
Nagarajan et al. (2016) [23]	Australia, Canada	Mixed-methods	Supervisor	Ex Phys, OT, Physio, SLP
Nagarajan et al. (2018) [24]	Australia, Canada	Mixed-methods	Supervisor	Ex Phys, OT, Physio, SLP
Panos (2005) [25]	US	Non-RCT	Student	SW
Reese et al. (2009) [26]	US	Non-RCT	Student	Psychology
Rule & Kelchner (2017) [27]	US, Kenya	Descriptive	-	SLP
Post-COVID articles				
Arundel et al. (2022) [28]	Canada	Qualitative	Supervisor	SW
Au et al. (2023) [29]	Canada	Mixed-methods	Student	SW
Bautista et al. (2020) [30]	US	Mixed-methods	Student	Pharmacy
Beiting & Nicolet (2020) [31]	US	Descriptive	-	SLP
Bowers et al. (2022) [32]	US	Mixed-methods	Supervisor	SLP
Chan et al. (2021) [33]	Australia	Descriptive	-	SLP
De Fries et al. (2021) [34]	US	Descriptive	-	SW
Easton et al. (2021) [5]	Australia	Qualitative	Supervisor	SLP
Fronek et al. (2021) [35]	Australia, NZ, US	Descriptive	-	SW
Furlong et al. (2021) [36]	Australia	Qualitative	Student	SLP
Gicas et al. (2021) [37]	Canada	Mixed-methods	Supervisor	Psychology
Gooding & Rushing (2022) [38]	US	Descriptive	-	Music therapy
Hatton et al. (2021) [39]	UK	Mixed-methods	Student	SW
Kourgiantakis et al. (2021) [40]	Canada	Descriptive	-	SW
MacKenzie et al. (2023) [41]	Canada	Mixed-methods	Supervisor	OT
Martin et al. (2022) [42]	Australia	Qualitative	Supervisor	Ex Phys, ND, OT, Physio, Rad, SLP, SW
Penman et al. (2022) [43]	Australia	Qualitative	Student + Supervisor	SLP
Salter et al. (2020) [3]	Australia	Descriptive	-	OT, Physio, SLP
Sheepway et al. (2023) [44]	Australia	Qualitative	Student	ND, OT, Podiatry, SLP
Short & Giles (2021) [45]	Australia	Descriptive	-	Medical radiation
Twogood et al. (2020) [6]	UK	Descriptive	-	Physio
Walker et al. (2022) [46]	Australia	Qualitative	Student	Ex Phys, ND, Physio, Psychology, SW
Whitehead et al. (2023) [47]	Australia	Qualitative	Student + Supervisor	OT, SLP
Zuchowski et al. (2021) [48]	Australia	Qualitative	Student, Supervisor + Placement Site	SW

*Abbreviations*: *Ex Phys*  Exercise Physiology, *ND*  Nutrition & Dietetics, *NZ*  New Zealand, *OT*  Occupational Therapy, *Physio*  Physiotherapy, *Rad*  Radiography, *RCT*  Randomised Control Trial, *SLP*  Speech & Language Pathology, *SW*  Social Work, *UK*  United Kingdom, *US*  United States

No randomised control trials (RCTs) were identified. Two non-RCTs compared telesupervision to in-person clinical supervision. Ten articles applied mixed-methods approaches, combining surveys and interviews with students (*n* = 4); supervisors (*n* = 2); and both students and supervisors (*n* = 4). Qualitative study designs were adopted in ten articles. Eleven articles described implementation of telesupervision.

Studies were conducted in Australia (*n* = 13), United States (*n* = 9), Canada (*n* = 5), and United Kingdom (*n* = 2). Cross-county collaborations were also reported (*n* = 4). Articles reported on clinical placements in rural areas (*n* = 17), metropolitan areas (*n* = 11), international contexts (*n* = 5), and simulated environments (*n* = 5). Chronologically, articles were divided into two groups: those prior to 2020 which focused on rural and international placements (*n* = 9); and articles from 2020 which explored responses to the COVID-19 pandemic (*n* = 24).

Telesupervision commonly involved students, supervisors, and clients in three separate locations (*n* = 15). Other configurations included the student onsite with clients receiving remote supervision (*n* = 7); mixed face-to-face and remote supervision (*n* = 5); and the supervisor onsite with clients, with students remotely located (*n* = 1). The data did not allow for the comparison of outcomes between the various configurations.

The articles included supervisors and students from a range of allied health disciplines as indicated in Table 2, most commonly speech-language pathology (*n* = 14), social work (*n* = 11), occupational therapy (*n* = 7), and physiotherapy (*n* = 6). Seven interprofessional placements were reported. Some mixed discipline articles included disciplines beyond the scope of this review: medicine (*n* = 3), nursing (*n* = 2), midwifery (*n* = 2), health science (*n* = 1), and education (*n* = 1). These data were removed where possible.

#### Outcomes derived from extracted quantitative data

Data were extracted from two quantitative articles (*n* = 2 non-RCTs) and seven mixed-methods articles (*n* = 7). Only seven of the ten mixed-methods articles reported quantitative findings to a sufficient level of detail for inclusion in this analysis. Most quantitative articles (*n* = 7) focused on student perspectives, with two articles reporting clinical supervisors’ perspectives [32, 37]. No articles reported objective data of student clinical competency development. Rather, quantitative measures primarily focused on self-reported ratings of satisfaction with telesupervision. Thus, in response to research question 1, evidence was not identified to directly support and guide educational providers in assuring quality telesupervision for allied health students.

Led by the data, the review explored research question 2 regarding student and clinical supervisor experience of telesupervision. Quantitative data was identified in relation to student and clinical supervisor satisfaction, reduction in student isolation, and development of student and supervisor skills.

Levels of student satisfaction with telesupervision were high. Psychology and social work students reported equal satisfaction with face-to-face supervision and telesupervision [25, 26], while psychology students reported that telesupervision had no significant impact on their ability to form supervisory relationships [26]. Students reported high levels of satisfaction with online supervisor availability, helpfulness and access to technology [29] as well as active engagement with their clinical supervisor, receiving feedback and feeling safe online [19]. Student satisfaction with online relationships also extended to interactions with other students [21].

Students consistently reported a reduction in their sense of isolation when using telesupervision or other forms of online communication with clinical supervisors or peers. Whilst this was particularly relevant to articles conducted during the COVID-19 pandemic, for example Hatton et al. [39], findings from articles published prior to the COVID-19 pandemic also highlighted this potential benefit for telesupervision (e.g., 25). Social work students who undertook international placements reported an immediate and dramatic drop in their sense of isolation (82%) when engaging in video-based telesupervision [25]. Similarly, 85% of occupational therapy students who attended placement in rural Queensland, Australia, reported video-based telesupervision to be an effective tool to reduce feelings of isolation [21]. During the COVID-19 pandemic, 83% of social work students surveyed reported the video-conferencing platform encouraged good conversations with their student peers [39].

Students also reported that the online telesupervision environment enabled them to develop specific clinical practice skills, including communication and teamwork [39], an increased sense of self-efficacy [26], confidence [30], and the opportunity to learn ‘real world skills’ for practice [39]. Approximately half of the surveyed students reported development of, or improved IT skills [29, 39], however problems with technology and internet connectivity for remote working and supervision remained a challenge for many [39]. In summary, students considered telesupervision an effective tool for remote allied health education and reported strongly positive experiences of telesupervision.

Challenges associated with the rapid move to telesupervision as a response to COVID-19 were noted in some articles, particularly related to students’ understanding of task expectations and ambiguity around task requirements [39]. While students reported high levels of satisfaction with access to technology, lower levels of satisfaction with technology training were recorded [29].

Reporting of supervisor experiences was conflicting with Gicas et al. [37] identifying that only 45% of clinical supervisors felt prepared for online clinical supervision, while Bowers et al. [32] reported that 71% of supervisors indicated that the transition to online telesupervision had no impact on supervisory abilities. These two articles, both from the COVID-19 transition phase were the only quantitative reports of supervisor experiences.

#### Outcomes derived from extracted qualitative data

Inductive content analysis of the extracted qualitative data derived six content categories: Participant connection, Structural support, Additional skill development, Quality, Transition, and Business as usual. Each category contained multiple subcategories of perspectives shared by both students and clinical supervisors. Unique clinical supervisor perspectives were identified across four sub-categories: Structural support, Additional skill development, Transition, and Business as usual. Unique student perspectives were identified across Quality, Transition, and Business as Usual sub-categories. Table 3 illustrates the six categories along with their sub-categories. Example data for each subcategory are provided in Appendix 2.

**Table 3:** Categories and subcategories identified in relation to clinical supervisors and student perspectives

Participant connection	Structural support	Additional skill development	Quality	Transition	Business as usual
Peer learning Value of connection Emotional safety & wellbeing	Infrastructure Processes Specific training Additional resources Time to establish^a^ Sufficient staff^a^	Technological Telehealth Reflective practice Interpersonal Ethics^a^	Enhanced placement learning Clinical supervisor effectiveness Meet standards	Struggle to adapt Online fatigue^a^ Problem solving^a^ Peer support^a^ Missed immersive experience^a^ Enjoyable^b^	Same benefit as face-to-face Less time consuming Authentic preparation for clinical work Face-to-face experiences still valuable^b^ Reduction of geographic barriers^a^

^a﻿^Clinical supervisors’ perspectives only; ^b^Students’ perspectives only

*Participant connection* related to peer learning among students, students’ need for increased emotional support, and the value of connection. Students and clinical supervisors recognised the importance of online peer learning among students, identifying the need for intentional provision of peer learning opportunities by supervisors [6] as well as those initiated by students [44]. Students and clinical supervisors valued face-to-face connection through hybrid placements as peer learning was acknowledged as more challenging through the online environment [47]. Several articles identified students’ increased need for intentional emotional support due to decreased face-to-face support [26, 28]. Authors explored meeting practical socioemotional needs of supervisors and students within telesupervision relationships, including an intentional focus on student safety and wellbeing, particularly when providing negative feedback to students [5].

*Structural supports* for telesupervision were identified by students and clinical supervisors. These included the need for effective telesupervision infrastructure including technology [26]; the establishment of processes that are responsive to the online environment [3, 47]; as well as the provision of specific training and resources to support telesupervision [46]. Unique to the clinical supervisor perspective were sub-categories related to the need for additional time to establish and set-up online placements [23], and increased staffing to manage the technical needs of telesupervision [5].

Students and clinical supervisors identified that engagement in telesupervision required *additional skill development*. This included the development of technological skills [6], telehealth skills [46], student reflective practice skills [48], and interpersonal skills within the online environment [49]. There was a sense of loss regarding the informal opportunities for connection between clinical supervisors and students afforded by face-to-face placements [5]. Clinical supervisors recognised skills required for telesupervision, including the development of their own reflective practice skills [5]; and online ethical practice, including confidentiality and privacy in online environments [3].

Three key elements relating to *quality* telesupervision were identified by clinical supervisors and students alike. Telesupervision was recognised as effective [21], resulting in students experiencing enhanced learning on placement due to opportunities for developing online capabilities [23]. To provide quality supervision of students, supervisors needed to be effective [47], with effectiveness related to the previous categories of connection, structural support, and the development of relevant skills. Finally, telesupervision practice needed to meet the professional standards relevant to the placement in order to meet the learning needs of the students [50].

In the *transition* to telesupervision, clinical supervisors and students identified the struggle to adapt to the new context [47]. Clinical supervisors missed the immersive experience of having students face-to-face [3], acknowledged the impact of online fatigue, and identified the need to plan online social activities [5]. In response to these challenges, they reported using problem-solving skills to support their transition to telesupervision [28], and the value of peer support for facilitating this transition [5]. Despite the initial struggle to adapt, students reported positive experiences of telesupervision [30].

*Business as usual* referred to the experiences of both students and clinical supervisors regarding their adaptation to the online environment. After a period of transition to telesupervision, both students and clinical supervisors discussed telesupervision providing the same overall benefit as more traditional or face-to-face supervision albeit using a different approach [46]. They reflected on telesupervision being less time-consuming once established [26]. Students and supervisors regarded telesupervision placements as authentic preparation for real-life clinical work, in which telehealth had become standard practice [43]. Students uniquely identified that telesupervision placements developed their understanding of how to support telehealth clients in the future [46]. Clinical supervisors also described telesupervision in relation to broadening geographic access for supervisors and students, including the benefits of a wider pool of clinical supervisors who could be recruited [43] and flexible access to students and clinical education tasks allowing switching between work roles or tasks [5]. While valuing telesupervision experiences, both students and clinical supervisors identified that they also valued face-to-face experiences, with students not wanting to miss out on these [43].

In addition to these six content categories were qualitative data that referred specifically to COVID-19 as an accelerant in the uptake of telesupervision and as an additional stressor on students and clinical supervisors. These references to COVID-19 were contextual to the publication period, rather than providing evidence that could support and guide the provision of telesupervision. Thus, a category relating to COVID-19 is not reported.

#### Synthesis of recommendations

Inductive content analysis of the extracted recommendations for telesupervision derived four categories: Intentional development and implementation of telesupervision, Student wellbeing and learning supports, Multi-level resourcing, and Responsive reimagining of clinical placement models.

A clear recommendation for *intentional development and implementation* of telesupervision emerged in the early studies [24, 26]. Acknowledgement of inherent differences between online and face-to-face spaces underpinned recommendations for deliberate development of online supervision, including structured time for clinical supervisor support and debriefing [5], alongside student support and peer mentoring strategies. Strategies to reduce the impact of online placements on students’ cultural and social experiences [44] were also recommended. A hybrid model of telesupervision alongside face-to-face supervision was recommended, with clear rationales for use of both modes (e.g., [26, 46, 47]).

*Student wellbeing and learning supports* were recommended as vital for quality telesupervision experiences. Flexible learning and mental health supports that are responsive to student accessibility needs, including remoteness, disability, socioeconomic situation, and lack of technology resources were recommended [29]. Further, consideration of students’ learning styles and personalities [47] was recommended alongside support for development of clinical skills and clinical reasoning [6, 48, 49]. Finally, deliberate development of professional skills through scaffolded reflective practice was recommended to enhance student learning [48].

*Multi-level resourcing* of online clinical placement was recommended, requiring investment of time and resources. Telesupervision required time for planning and modelling problem-solving strategies. Additional training for clinical supervisors was critical to ensuring high quality experiences [32]. When planning clinical placement using telesupervision, responsivity to the needs and priorities of placement sites was key [37]. Universities were challenged to invest in reciprocal relationships with communities for the purpose of developing mutually beneficial telesupervison experiences [3]. To ensure the effectiveness of telesupervision technology, barriers needed to be addressed [23, 24, 40] including student, clinical supervisor, and client familiarity with the relevant technology [23]. More broadly, student and clinical supervisor training regarding virtual supervision processes and relevant technology optimised student learning [19] and promoted development of supervisor confidence and competence [31].

Universities were encouraged in *responsive reimagining of allied health clinical placement models* through continued and expanded use of telesupervision. Integration of telesupervision into future clinical education could enhance flexibility for supervisors, increase client services, and expand student learning opportunities [3]. While this reimagining should creatively consider the role of location and space [28] it must also comply with university and professional governance structures [27]. The need for an integrated cross-sector approach including universities, clinical supervisors, governance and funding bodies was identified [34].

### Findings from professional document review

In total, 46 websites were searched. Of these, only the Health Education and Training Institute (HETI) website returned a result from the primary keyword *telesupervision*. Visual review of the HETI link identified no content, and no downloadable profession-specific or organisation-based documents related to telesupervision. Manual following of additional links located on the page also yielded no content related to telesupervision.

All websites returned multiple results from the additional keywords *clinical education*,* student supervision*,* student placement*,* telehealth*,* telepractice*, and *student learning.* Visual review of search results and exploration of links yielded fifteen potentially relevant items based on the content or document title. Closer review identified six items referring to telesupervision (refer to Table 4). Two websites, ClinEdAus and Speech Pathology Australia (SPA), contained formal content without downloadable documents. Three websites included downloadable documents, with two relevant documents sourced from Australian Collaborative Education Network (ACEN; renamed WIL Australia), and one relevant document each from Audiology Australia, and HETI.

**Table 4:** Website content and profession/organisation-based documents identified

Document/Webpage	Resource type	Intended audience	Discipline
Australian Collaborative Education Network [51]	Guidelines	Universities, clinicians & supervisors	N/A
Australian Collaborative Education Network [52]	Guidelines	Industry & community partners	N/A
ClinEdAus [53]	Webpage content	Clinical supervisors	Allied health
Audiology Australia [54]	Guidelines	Clinicians	Audiology & audiometry
Speech Pathology Australia [55]	Webpage content	Universities	Speech pathology
Health Education and Training Institute [56]	Literature review	Universities, clinicians & supervisors	Health (including nursing & medicine)

#### Summary of website content and profession-based documents

Due to the low yield, a summary of content, rather than content analysis, is provided. Sources of information are presented in order of most to least informative. Notably, no policy or accreditation documents were identified.

The ACEN website contained two WIL guides, designed for practitioners [51] as well as industry and community partners [52] respectively. These guides address the planning, creation, and support of online WIL experiences across disciplines and contexts. While the breadth included allied health clinical placements, specific guidance for this context was not provided. The ClinEdAus website provided a brief overview “Virtual Supervision and Virtual Placements” [53], limited to an infographic of models, advantages and challenges, a planning checklist for supervisors, and additional resources.

Audiology Australia [54] and Speech Pathology Australia [55] acknowledged the need to consider innovative clinical supervision models, including telesupervision, but provided no practical guidance for the implementation of telesupervision. Speech Pathology Australia placed limitations on the use of telesupervision within speech pathology WIL, stating “Further, while SPA supports telepractice as an alternative service delivery model, at a minimum, student speech pathologists are required to provide *in-person*,* face-to-face services during their penultimate or final placement*” [55].

HETI published a literature review of “Curriculum Interventions and pedagogical approaches for virtual care” [56], focused on virtual care delivery and how to build capacity for virtual care in “current and future clinicians” (p. [5]). However, this focused on the existing workforce, and content related to clinical placements, student training, and consequently future clinicians was limited.

## Discussion

This scoping review was driven by the current higher education and health care contexts in which telesupervision has returned from the emergency protocols of COVID back to everyday practice [14]. It aimed to provide a foundation for building an evidence-base to support scholarly use of telesupervision in clinical education of allied health students. The findings are discussed in relation to the three research questions below.

### Evidence-based guidance for assuring quality telesupervision

Quality in WIL is increasingly defined by quality frameworks and professional accreditation standards which require demonstration of scholarly, or evidence-based rationales to support innovation [56]. This review has established that the published research is not of sufficient strength to support and guide educational providers in assuring quality telesupervision for allied health students. The published evidence is limited to surveys of student and clinical supervisor satisfaction; qualitative experiences and perceived outcomes of telesupervison; and descriptions from the rapid COVID-19 pivot. It does not directly support use of telesupervision as a tool to ensure student learning as required in the context of WIL Quality Frameworks and professional accreditation bodies [9, 55].

### Evidence regarding supervisors’ and allied health students’ experiences

Despite this paucity of empirical evidence around educational outcomes, the review of the literature found clear evidence of high student and clinical supervisor satisfaction within telesupervision placements [30]. Importantly, this included a sense among clinical supervisors of providing students with the same benefit as a face-to-face placement, but through a different approach [46]. Clinical supervisors and students also identified the development of valuable skills for working in telehealth contexts through the experience of telesupervision [46], facilitating students’ job readiness with skills and experience relevant to current practice. Key to this experience was the connection between students and clinical supervisors, through formal and informal peer learning alongside the intentional creation of emotional safety and wellbeing [10].

Overall, findings based on clinical supervisor and student perspectives recommended a hybrid approach to the use of telesupervision alongside face-to-face supervision across a student’s university course. Based on their experiences, students and clinical supervisors referred to ‘quality’ telesupervision in relation to: Enhanced student learning outcomes, the effectiveness of supervision in supporting these outcomes, and meeting required standards. While no studies have applied these data-driven descriptors to evaluate telesupervision, they may prove useful concepts as a foundation for future evaluative research.

Though clinical supervisors and students reported enjoying the affordances of telesupervision, they also reported missing the face-to-face connection that had experienced on earlier placements [3]. Student and supervisor familiarity with a range of online tools scaffolded both formal and informal learning opportunities, however, a need to intentionally support transition to the online environment was also identified, including specific training in technology and telehealth skills for both students and clinical supervisors, as well as ethical practice and problem solving for these new environments [46]. These tensions highlight the need for intentional scaffolding of telesupervision in the curriculum and provision of training and infrastructure support for supervisors. In addition, the need for intentional development of multi-level structural support as a foundation for telesupervision also emerged strongly [23, 29].

### Professional requirements and/or information to support and guide the provision of telesupervision in Australia

The review of Australian-based professional documents reflected the lack of evidence available to support and guide educators in assuring quality telesupervision and meeting the learning needs of allied health students [50]. For example, while WIL Australia [51, 52] provided overarching guidelines for telesupervision, consideration of telesupervision in the allied health context and application within specific professions was significantly lacking. Guidance was only identified within one profession, and this guidance focused on restricting telesupervision across a students’ professional education [57] rather than supporting quality telesupervision. Based on the available evidence-base, challenging this stance towards limitation of telesupervision in a post-COVID19 context is difficult. Student and clinical supervisor experiences are not sufficiently robust to assure quality telesupervision that meets the needs quality frameworks and accreditation requirements.

### Implications

Consistent across the reviewed literature was the capacity for telesupervision to open opportunities for clinical placements which are inaccessible for traditional face-to-face models. Findings demonstrate that telesupervision can provide a positive path to broadening geographic access to quality learning experiences for both supervisors and students, which is critical for workforce development [58]. This includes rural and remote placements [57] where full-time onsite discipline specific supervisors may not be available, a practice which dates back to before COVID-19 when telesupervision was largely used for rural, remote and international placements.

This review supports continued deliberate integration of telesupervision as a valuable WIL model in allied health education curricula and highlighted the need to build on the emerging evidence to guide the development and implementation of high-quality allied health telesupervision practices. Telesupervision offers benefits that complement face-to-face clinical supervision models, and its ongoing use will ensure that students are well prepared for contemporary healthcare models including telehealth. Acknowledgement of inherent differences between online and face-to-face environments underpinned recommendations for intentional resourcing and development of telesupervision within universities, health providers, and allied health professions.

Further research regarding the efficacy of telesupervision is needed as a foundation for developing guidelines for telesupervision including the use of telesupervision in a hybrid context. This can form the basis for appropriate governance and infrastructure required to ensure quality. Evidence will also support allied health professionals, health services and accrediting bodies that currently rely on largely descriptive articles and anecdotal evidence when determining ‘if,’ how much,’ or ‘what type of’ telesupervision is acceptable during student clinical placements.

### Limitations

The limitations of this review must be acknowledged. As this is a scoping review, the analysis is thematic and narrative in nature. Critical appraisal of each included article was not undertaken. Identified articles were primarily descriptive, with few identified articles adopting rigorous research methodologies. Notably, there has been limited direct comparison between telesupervision and face-to-face supervision, and limited evaluation of educational outcomes achieved following telesupervision. This review relied on two controlled studies published almost two decades ago [25, 26] as the only comparators of telesupervision and face-to-face supervision. Limited review of the grey literature is also acknowledged. An international review of literature such as unpublished reports may give further context to the Australian professional document review provided.

## Conclusion

This review provides a foundation for future application of telesupervision within allied health education curricula, identifying a need for ongoing research into the effectiveness of telesupervision in quality student learning. The findings challenge views of telesupervision as supplementary, or “second-best” by demonstrating that telesupervision was a responsive clinical placement model that was experienced positively by both students and clinical supervisors, while supporting the creation of a telepractice ready workforce. We are yet to see if telesupervision and the benefits it offers remain part of every student’s training in the post-pandemic era, or if it is once again relegated to rural, remote, and international clinical placements. However, this review clearly illustrated that if telesupervision is to become “usual practice,” a stronger evidence-base alongside evidence-based professional development for clinical supervisors transitioning to telesupervision will be central to this.

## Supplementary Information

Supplementary Material 1.

## Data Availability

All data generated or analysed during this study are available on request.
