# Supplementary material for: Telesupervision of allied health students during work-integrated learning: a scoping review of the international evidence-base and its representation in localised professional guidelines
Source: BMC Med Educ. 2026 Aug 6;26:1767. doi: 10.1186/s12909-026-09440-z (PMC13729410; doi:10.1186/s12909-026-09440-z)
Supplement: Supplementary file 1 [file 12909_2026_9440_MOESM1_ESM.docx]

**Appendices**

***Appendix 1 Example search concept definitions***

| **Ebscohost**  Search Field: Select a field (optional) ie searches Title, abstract, subject headings  Peer reviewed, All source types, 2002-2023, Expanders – Apply equivalent subjects | |
| --- | --- |
| **1** | **Concept: Student** |
|  | ("allied health" OR health OR college OR University OR undergraduate OR postgraduate) student |
| **2** | **Concept: Learning context** |
|  | (clinical OR practice) placement) OR "workplace learning" OR "work-integrated learning" OR "Clinical experience" OR (SU "CLINICAL education") |
| **3** | **Concept: Telesupervision** |
|  | ( (SU "Telemedicine") OR (SU "Clinical Supervision") OR e-supervision OR telesupervision OR ((virtual OR online OR remote) supervis*) OR "clinical educator" OR teleconferenc* ) OR telepractice |
| **4** | **Combined Concepts: Student AND Learning context** |
|  | ( ("allied health" OR health OR college OR University OR undergraduate OR postgraduate) student ) AND ( (clinical OR practice) placement) OR "workplace learning" OR "work-integrated learning" OR "Clinical experience" OR (SU "CLINICAL education") ) |
| **5** | **Combined Concept: Learning context AND Telesupervision** |
|  | ( (clinical OR practice) placement) OR "workplace learning" OR "work-integrated learning" OR "Clinical experience" OR (SU "CLINICAL education") ) AND ( ( (SU "Telemedicine") OR (SU "Clinical Supervision") OR e-supervision OR telesupervision OR ((virtual OR online OR remote) supervis*) OR "clinical educat*" OR teleconferenc* ) OR telepractice ) |
| **6** | **Combined Concept: Student AND Learning context AND Telesupervision** |
|  | ( ("allied health" OR health OR college OR University OR undergraduate OR postgraduate) student ) AND ( (clinical OR practice) placement) OR "workplace learning" OR "work-integrated learning" OR "Clinical experience" OR (SU "CLINICAL education") ) AND ( ( (SU "Telemedicine") OR (SU "Clinical Supervision") OR e-supervision OR telesupervision OR ((virtual OR online OR remote) supervis*) OR "clinical educator" OR teleconferenc* ) OR telepractice ) |

***Appendix 2 Qualitative data analysis: Categories, subcategories and examples***

| Category | Subcategory | Examples from extracted data | Article |
| --- | --- | --- | --- |
| Participant connection | Peer learning | Connecting students from different sites with each other so they could engage in some peer learning activities | Chipchase et al. (2014) |
|  | Value of connection | Supervisory relationship reported to be comfortable but could be important to establish relationship prior to videoconferencing supervision, as reported some emotional elements were lost in this format. | Reese et al. (2009) |
|  | Emotional safety & wellbeing | Student safety related specifically to Covid-19. There were 2 aspects to this (a) physical safety/protection from the disease & (2) emotional/mental safety in the rapidly changing workforce …Student anxiety & wellbeing was also linked specifically with Covid-19 in terms of health (their own & family members) and then the potential impact on placement and academic progression… | Fronek et al. (2021) |
| Structural support | Infrastructure | Videoconferencing concerns and recommendations - positive comments about videoconferencing were tempered with concerns about technology needing to be reliable. | Reese et al. (2009) |
|  | Processes | Key considerations, processes, and suggestions for communicating and providing feedback to students; increased scheduling of specific feedback times, increased level of written feedback & student reflection | Whitehead (2023) |
|  | Specific training | Future considerations for the success of the remote supervision model: need for … development of training resources for CEs, and opportunity for CEs and students to learn new skills in technology use. | Whitehead (2023) |
|  | Additional resources | Challenges, limitations and the need for resources, training and support for clients as well as students and clinicians. | Walker et al. (2022) |
|  | Time to establish | From a Placement Coordinator perspective, setting up and supporting the online placements involved more work and time than a standard service-learning placement, although once the initial measures were implemented, efficiencies were gained with repetition | Salter et al. (2020). |
|  | Sufficient staff | Supervisors can't have an ear on multiple student groups at a time - this resulted in a smaller staff to student ration, increasing the cost of the placement. | Easton et al. (2021) |
| Additional skill development | Technological | students acknowledged growth in their technological awareness and felt well-placed to use technology in their future work to improve productivity and support rural health service delivery. | Sheepway et al. (2023) |
|  | Telehealth | Coming away with a new set of skills in telehealth | Walker et al. (2022) |
|  | Reflective practice | Supervisors described continuously reflecting on their practice during and after the placement, including how to best adapt their teaching and the student learning environment to the online mode | Easton et al. (2021) |
|  | Interpersonal | Heightened focus on communication skills in a telepractice environment; the need to provide clear instructions and feedback provided to clients, and the value of this being timely, specific, and clearly articulated; perception of needing to be more animated to keep clients engaged was reported by some | Furlong et al. (2021) |
|  | Ethics | Students needed to ensure privacy and confidentiality measures were adhered to in their homes as well as across multiple online platforms, which was sometimes challenging. | Salter et al., (2020) |
| Quality | Enhanced placement learning | University clinical education coordinator perceived benefits: enhanced learning experience for students on placements (perceived benefit to students) | Nagarajan et al. (2016) |
|  | Clinical supervisor effectiveness | Facilitator attributes influenced the effectiveness of VC for learning and support | Furness et al. (2015) |
|  | Meet standards | Students were able to complete their placements during high rates of placement cancellation. Students passed their curriculum and clinical learning needs whilst contributing positively to clients and organizations. | Salter et al. (2020) |
| Transition | Struggle to adapt | Difficulties providing an accurate assessment of students' progress, supervisors' inherent struggle to provide a field education experience that was nontraditional in every way | Arundel et al. (2022) |
|  | Online fatigue | Additional time spent online could add to the feeling of "online fatigue". | Easton et al. (2021) |
|  | Problem solving | Supervisors viewed flexibility and problem-solving skills as fundamental for transitioning to the online telesupervision modality. | Easton et al. (2021) |
|  | Peer support | Support provided to clinicians in mentoring virtual students. Support was provided through Microsoft Teams meetings to provide a forum for question and discussion. These were recorded and circulated to all mentors afterwards. Role modelling was also vital | Twogood (2020) |
|  | Missed immersive experience | Challenges: limited opportunities for students to benefit from the immersive experience | Salter et al (2020) |
|  | Enjoyable | Online simulation-based learning was a positive experience | Penman et al. (2022) |
| Business as usual | Same benefit as face to face | Quality interprofessional education experiences can be achieved in telehealth | Walker et al (2022) |
|  | Less time consuming | Videoconferencing was additionally found to be more efficient and goal-directed because of the format (less social chit-chat). | Reese et al. (2009) |
|  | Authentic preparation for clinical work | Participating students identified that the placement helped strengthen their social work skills and assisted the community during the COVID-19 crisis." | Zuchowski (2021) |
|  | Face-to-face experiences still valuable | Although online and in-person simulation-based learning are comparable, in-person experiences are still valued | Penman et al. (2021) |
|  | Reduction of geographic barriers | The virtual nature of the placement enabled students with pre-existing health conditions or living abroad to complete clinical placement and thus continue with their studies. | Twogood (2020) |

***Appendix 3 Identified Professional Associations, Boards and Supporting Organisations***

| Discipline | Organisation | Website |
| --- | --- | --- |
| *AHPRA regulated disciplines* |  |  |
| Aboriginal and Torres Strait Islander Health Practitioner | National Association of Aboriginal and Torres Strait Islander Health Workers and Professionals | <https://www.naatsihwp.org.au/> |
|  | AHPRA - Aboriginal and Torres Strait Islander Health Practice Board of Australia | <https://www.atsihealthpracticeboard.gov.au/> |
| Chinese Medicine | Australian Acupuncture and Chinese Medicine Association | <https://www.acupuncture.org.au/> |
|  | AHPRA - Chinese Medicine Board | <https://www.chinesemedicineboard.gov.au/> |
| Chiropractic | Australian Chiropractors Association | <https://www.chiro.org.au/> |
|  | Chiropractic Australia | <https://chiropracticaustralia.org.au/> |
|  | AHPRA – Chiropractic Board of Australia | [https://www.chiropracticboard.gov.au/](https://www.chiropracticboard.gov.au/ ) |
| Dentistry | Australian Dental Association | <https://www.ada.org.au/> |
|  | AHPRA - Dental Board of Australia | <https://www.dentalboard.gov.au/> |
| Medical Radiation | Australian Society of Medical Imaging and Radiation Therapy | <https://www.asmirt.org/> |
|  | AHPRA – Medical Radiation Practice Board of Australia | <https://www.medicalradiationpracticeboard.gov.au/> |
| Occupational Therapy | Occupational Therapy Australia | <https://otaus.com.au/> |
|  | AHPRA - Occupational Therapy Board of Australia | <https://www.occupationaltherapyboard.gov.au/> |
| Optometry | Optometry Australia | <https://www.optometry.org.au/> |
|  | AHPRA – Optometry Board of Australia | <https://www.optometryboard.gov.au/> |
| Osteopathy | Osteopathy Australia | <https://www.osteopathy.org.au/> |
|  | AHPRA – Osteopathy Board of Australia | <https://www.osteopathyboard.gov.au/> |
| Paramedicine | Australian College of Paramedicine | <https://paramedics.org/> |
|  | AHPRA – Paramedicine Board of Australia | <https://www.paramedicineboard.gov.au/> |
| Pharmacy | Pharmaceutical Society of Australia | <https://www.psa.org.au/> |
|  | AHPRA – Pharmacy Board of Australia | <https://www.pharmacyboard.gov.au/> |
| Physiotherapy | Australian Physiotherapy Association | <https://australian.physio/> |
|  | AHPRA – Physiotherapy Board of Australia | <https://www.physiotherapyboard.gov.au/> |
| Podiatry | Australian Podiatry Association | <https://www.podiatry.org.au/> |
|  | AHPRA – Podiatry Board of Australia | <https://www.podiatryboard.gov.au/> |
| Psychology | Australian Psychological Society | <https://psychology.org.au/> |
|  | AHPRA – Psychology Board of Australia | <https://www.psychologyboard.gov.au/> |
| Self-regulating disciplines |  |  |
| Audiology | Audiology Australia | <https://audiology.asn.au/Home> |
| Dietetics | Dietitians Australia | <https://dietitiansaustralia.org.au/> |
| Exercise and sports science | Exercise and Sports Science Australia | <https://www.essa.org.au/> |
| Genetic counselling | Australasian Society of Genetic Counsellors (under the auspices of Human Genetic Society of Australasia) | <https://www.hgsa.org.au/asgc> |
| Music therapy | Australian Music Therapy Association | <https://www.austmta.org.au/> |
| Orthotics & prosthetics | The Australian Orthotic Prosthetic Association | <https://www.aopa.org.au/> |
| Perfusionists | Australian and New Zealand College of Perfusionists | <https://anzcp.org/> |
| Rehabilitation counselling | Australian Society of Rehabilitation Counsellors Ltd. (ASORC) | <https://www.asorc.org.au/> |
| Social work | Australian Association of Social Workers | <https://www.aasw.asn.au/> |
| Speech pathology | Speech Pathology Australia | <https://www.speechpathologyaustralia.org.au/> |
| Generic allied health and educational bodies or organisations | | |
|  | Health Education and Training Institute (HETI) | <https://www.heti.nsw.gov.au/about-heti> |
|  | Services for Australian Rural and Remote Allied Health (SARRAH) | <https://sarrah.org.au/> |
|  | Indigenous Allied Health (IAHA) | <https://iaha.com.au/> |
|  | Allied Health Professions Australia (AHPA) | <https://ahpa.com.au/> |
|  | ClinEdAus | <https://www.clinedaus.org.au/> |
|  | Australian Collaborative Education Network (ACEN) | <https://acen.edu.au/> |
